# Evaluation of carcass quality, body and pulmonary lesions detected at the abattoir in heavy pigs subjected or not to tail docking

**DOI:** 10.1186/s40813-022-00297-4

**Published:** 2023-02-15

**Authors:** Laura Amatucci, Diana Luise, Andrea Luppi, Sara Virdis, Alice Prosperi, Agatha Cirelli, Claudia Bosco, Paolo Trevisi

**Affiliations:** 1 Department of Agricultural and Food Sciences (DISTAL), Alma Mater Studiorum-University of Bologna, Viale G. Fanin 46, 40127 Bologna, Italy; 2 Istituto Zooprofilattico Sperimentale della Lombardia e dell’Emilia Romagna (IZSLER), “Bruno Ubertini”-Via Bianchi, 9, 25124 Brescia, Italy

**Keywords:** Pig, Protected Designation of Origin, Tail docking, Transport, Season

## Abstract

**Background:**

Nowadays, body and tail lesions and respiratory disease are some of the greatest problems affecting the health and welfare of pigs. The aim of the study was to measure the prevalence of pleurisy, bronchopneumonia (enzootic pneumonia like lesions) and lesions on tail and body of heavy pigs subjected or not to tail docking through the inspection in Italian abattoirs. Additionally, the effect of tail docking and season was investigated on carcass quality (weight, % of lean meat, and Protected Designation of Origin (PDO) classification). For this purpose, a total 17.256 carcasses belonging to 171 batches from 103 farms were inspected in an Italian abattoir between 2019 and 2022. Enzootic pneumonia (EP) like lesions were scored according to the Madec and Derrien method, while pleurisy was scored using the Italian Slaughterhouse pleuritic evaluation system (SPES). For the tail and body, the lesions were scored according to Welfare Quality. The lesion score index (LSI) was calculated for each area. Data were analysed using a general linear model (GLM) including tail caudectomy, season and distance of the farm from the abattoir.

**Results:**

The warm season increased the percentage of lesions in carcasses in all parts of the body observed (*P* < 0.0001). The presence of undocked tail increased the LSI of the tail (*P* < 0.0001). The percentage of limbs lesions with score 2 and limbs LSI increase with increasing duration of transport (coef. = 0.003, *P* < 0.001; coef. = 0.008, *P* < 0.001; respectively). The hot carcass weight and the percentage of carcasses included in the PDO were higher in batches with docked tails (*P* = 0.027; *P* < 0.001, respectively), while the percentage of lean meat was higher in batches with undocked tails (*P* < 0.001). There was a negative correlation between the percentage of carcasses included in PDO and the LSI of tail (r = − 0.422; *P* < 0.001).

**Conclusions:**

In conclusion, the presence of the undocked tail and the warm season can be considered risk factors for the prevalence of tail lesions, while long transport can increase limb lesions. Furthermore, the carcass weight and meat quality were negatively influenced by tail lesions.

**Supplementary Information:**

The online version contains supplementary material available at 10.1186/s40813-022-00297-4.

## Background

One of the aims of carcass inspection at the abattoir is to monitor possible zoonotic infections and to assess the presence of animal diseases even if they have no consequences on the hygienic quality of the meat and therefore on its placing on the market [1]. The evaluation of lung lesions and pleurisy at the abattoir is an important monitoring tool as it allows to assess the incidence and severity of these lesions, and it can also let to estimate the economic impact of respiratory diseases on the porcine production chain, as well as their serious impact on animal health and welfare [2]. Respiratory diseases of swine can be due to different factors including infections by primary and secondary pathogens, host and environmental conditions. One of the most important respiratory diseases in pig production is the enzootic pneumonia, caused by *Mycoplasma hyopneumoniae* [2, 3]. It is normally characterized by bronchopneumonia in cranio-ventral lobes of the lung. Pleuritis can be cranio-ventral or dorso-caudal and the border between the two areas is lined by the dorsal endpoints of interlobar fissures. Pleural lesions, involving dorso-caudal lobes, are normally attributable to *Actinobacillus pleuropneumoniae* [4].

Furthermore, the observation of carcass injuries at the abattoir is considered a retrospective opportunity to evaluate the welfare status of animals prior to their arrival at the abattoir. Nowadays, tail lesions are considered one of the main welfare problems in pigs [5]. They usually result from behavioural problems that are caused or fostered by stressful conditions, so they are considered one of the key welfare indicators in pig farming [5–7]. Several factors can affect the welfare status of pigs, and among them, seasons and temperature are known to influence body and tail lesions even during breeding than during transport [8, 9]. Indeed, when the temperature is out of the comfort zone, it can increase the stress of pigs and then favour the presence of aggressive behaviours [8]. In addition, it has been observed that the position of pigs inside the truck during transport can also change with temperature and as a consequence the probability of body lesions [9, 10].

The transport to the abattoir is a dangerous situation for the health of the animals, in particular when the driving condition, driving style and suspension are not optimal and can cause vibration and loss of balance in the animals [11, 12]. Some studies reported that the length of the journey can influence blood parameters and the mortality of animals [13, 14]. Both respiratory diseases and tail injuries are associated with the reduction of performance in pigs [15]. In addition, tail lesions, especially severe ones, are associated with carcass condemnation, trimming and the reduction of carcass weight [15]. Tail biting has also been associated with other problems which derive from tail infections, such as spinal abscesses and pyaemias in different parts of the body, which, in addition to leading to a reduced growth rate, can lead to the total exclusion of the carcass from the market [5, 16].

Few studies have found an association between tail lesions and pleurisy, pneumonia, and pleuropneumonia by observation at the abattoir [17], while other studies did not find any association between these factors [15]. The introduction of restrictions in tail docking, seems to be associated with a rise on tail lesion, however, it is not clear if this decision can increase the risk of carcass defects and reduced health.

The hypothesis was that the presence of undocked tail versus docked tail can increase the frequency and severity of tail lesions, the risk of pulmonary lesions and therefore the carcass quality.

The objective of this study was to evaluate, at the abattoir, the prevalence and severity of lung lesions, pleurisy and lesions on the body as well as to assess the effect of caudectomy, slaughter season and the distance of the farm to the abattoir on the prevalence of external lesions, pneumonia, pleurisy and carcass quality assessed as inclusion on the Protected Designation of Origin (PDO) chain. Finally, the correlation between the presence of tail lesions, pleurisy and pneumonia and inclusion on PDO of the carcass was explored.

## Results

### Data and descriptive statistics

To test the hypothesis, a total 18.976 carcasses belonging to 151 batches from 94 farms were scored in an Italian abattoir between 2019 and 2022. The results of the descriptive analysis for carcass quality and body lesions are shown in Table 1. The mean of kilometres of distance from farm to abattoir was 101.65 km for tail docked batches and 110.83 km for tail undocked batches. The average hot carcass weight was 141.15 kg in pigs with docked tail and 138.69 kg in pigs with undocked tail, while lean meat percentage was 52.16% and 53.36% respectively.

**Table 1:** Descriptive analysis of lesions in various parts of the body

Item	N batches		Mean		StDev		Minimum		Maximum	
	Docked	Undocked	Docked	Undocked	Docked	Undocked	Docked	Undocked	Docked	Undocked
Km abattoir	143	28	101.65	110.83	78.44	109.14	15.70	20.30	394.40	322.60
Average hot carcass weight, kg	73	27	141.15	138.69	5.59	6.76	121.01	119.91	150.48	152.31
Average cold carcass weight, kg	143	27	139.21	135.91	6.12	6.62	118.58	117.51	153.35	149.26
Average % lean meat	143	27	52.16	53.36	1.40	1.34	48.08	51.71	54.98	56.98
N animals per batch	143	28	100.72	101.89	21.58	39.62	23.00	17.00	154.00	143.00
Lesions ears 0, %	143	28	89.86	92.43	12.27	6.33	3.17	79.03	100.00	100.00
Lesions ears 1, %	143	28	6.29	5.83	10.06	6.03	0.00	0.00	96.83	19.35
Lesions ears 2, %	143	28	3.84	1.74	4.96	2.33	0.00	0.00	23.64	8.70
LSI ears^1,2^	143	28	13.98	9.32	15.75	7.40	0.00	0.00	96.83	22.58
Lesions forequarter 0, %	143	28	80.73	86.35	12.67	10.17	44.00	61.54	98.29	100.00
Lesions forequarter 1, %	143	28	11.13	9.43	7.29	8.32	0.00	0.00	35.00	31.03
Lesions forequarter 2, %	143	28	8.15	4.22	7.77	4.45	0.00	0.00	32.00	15.22
LSI forequarter^1,2^	143	28	27.44	17.87	19.73	13.32	2.56	0.00	84.00	46.15
Lesions trunk 0, %	143	28	82.05	83.26	10.75	13.04	41.00	50.77	100.00	98.68
Lesions trunk 1, %	143	28	11.89	11.80	7.51	9.79	0.00	0.00	34.15	36.92
Lesions trunk 2, %	143	28	5.80	4.95	5.69	4.94	0.00	0.00	36.00	18.97
LSI trunk^1,2^	143	28	23.49	21.69	15.56	17.12	0.00	2.36	95.00	63.79
Lesions hindquarter 0, %	143	28	92.59	93.00	6.31	6.51	63.00	74.14	100.00	100.00
Lesions hindquarter 1, %	143	28	5.35	5.50	4.87	5.29	0.00	0.00	28.46	20.69
Lesions hindquarter 2, %	143	28	2.05	1.56	2.76	2.08	0.00	0.00	16.67	8.00
LSI hindquarter^1,2^	143	28	9.46	8.61	8.42	8.15	0.00	0.00	50.00	31.03
Lesions limbs 0, %	143	28	98.95	98.28	1.79	1.95	89.63	93.10	100.00	100.00
Lesions limbs 1, %	143	28	0.76	1.33	1.63	2.01	0.00	0.00	10.37	6.90
Lesions limbs 2, %	143	28	0.27	0.39	0.66	0.70	0.00	0.00	4.90	2.36
LSI limbs^1,2^	143	28	1.31	2.11	2.18	2.13	0.00	0.00	10.37	6.90
Lesions tail 0, %	143	28	70.44	4.96	16.57	14.06	0.00	0.00	99.18	73.08
Lesions tail 1, %	143	28	23.01	27.27	12.72	19.51	0.00	0.00	57.69	81.62
Lesions tail 2, %	143	28	7.08	67.77	11.60	24.14	0.00	0.00	80.00	100.00
LSI tail^1,2^	143	28	37.17	162.80	27.14	34.36	0.82	40.00	180.00	200.00

^1^LSI: lesion score index

^2^Values are calculated on a range from 0 to 200 considering the prevalence and severity of lesions, where 0 is absence and 200 all carcasses in the batch show severe lesions

The frequency of ear lesions of score 0 was 89.86% for pigs with docked tails and 92.43% for pigs with undocked tails. The percentage of ear lesions of scores 1 and 2 was 6.29% and 3.84% in docked tails and 5.83% and 1.74% for pigs with undocked tails. The LSI of ears was 13.98 and 9.32 in pigs with docked and undocked tails respectively. The percentage of lesions with score 0 in forequarter was 80.73% and 86.35% in pigs with docked and undocked tail respectively, while the mean of lesions of scores 1 and 2 were 11.13% and 8.15% for docked tails and 9.43% and 4.22% for undocked tails. The LSI of forequarter was 27.44 in docked tails and 17.87 in undocked tails. The mean of lesions of trunk of score 0 was 82.05% for pigs with docked tails and 83.26% for pigs with undocked tails. The LSI of trunk was 23.49 for pigs with docked tails and 21.69 in pigs with undocked tails. For hindquarter lesions of score 0 was 92.59% for docked tails and 93.00% in undocked tails, while the LSI was 9.46 and 8.6, respectively. The percentage of LSI of limbs was 1.31 for docked tails and 2.11 for undocked tails. The percentage of score 0 for tails was 70.44% in docked tails and 4.96% undocked tails, while the LSI was respectively of 37.17 for docked tails and 162.8 for undocked tails.

The results of the descriptive analysis of the batches by indices and prevalence of lung and pleural injuries are shown in Table 2. Lungs were analyzed from 128 batches with docked tails and 19 batches with intact tails. Bronchopneumonic lesions suggestive of enzootic pneumonia (EP-like) considering all the batches were detected in 27.32% of the lungs scored, and the EP-like average value was 0.89. The percentage and average values of EP-like lesions were 28% and 0.89 in tail docked batches and 26.64% and 0.90 in tail undocked batches. The average value of the pleuritis at the abattoir was evaluated using the Slaughterhouse pleuritis evaluation system (SPES) was 0.79 and 0.84 in tails docked and undocked tails and the percentage of pleurisy was respectively 36.11% and 40.47% in tails docked and undocked tails. The *Actinobacillus pleuropneumoniae* index (APPI) was 0.66 in tails docked and 0.67 in undocked tails (mean: 0.67), and the percentage of scores > 2 was 23.28% in batches with docked tails and 23.35% in batches with undocked tails.

**Table 2:** Descriptive analysis of indexes and prevalence of lung lesions and pleuritis

Item	N batches		Mean		StDev		Minimum		Maximum	
	Docked	Undocked	Docked	Undocked	Docked	Undocked	Docked	Undocked	Docked	Undocked
Km abattoir	128	19	103.76	122.06	75.76	110.36	16.30	20.30	394.40	314.00
Average hot carcass weight, kg	58	18	141.25	137.27	5.25	7.35	126.04	119.91	150.48	149.79
Average cold carcass weight, kg	128	18	139.36	134.52	6.05	7.21	123.51	117.51	153.35	146.79
Average lean meat, %	128	18	52.06	53.66	1.41	1.49	48.08	51.71	54.98	56.98
N animals per batch	128	19	94.77	95.37	23.78	40.53	23.00	42.00	236.00	223.00
Madec	128	19	0.89	0.90	0.80	0.89	0.00	0.03	4.87	4.06
Lungs lesions, %	128	19	28.00	26.64	15.40	15.10	0.00	2.99	74.56	57.14
Spes	128	19	0.79	0.84	0.43	0.29	0.00	0.38	1.93	1.42
Pleurisy, %	128	19	36.11	40.47	17.75	11.09	0.00	23.53	85.00	58.74
Appi index	128	19	0.66	0.67	0.42	0.29	0.00	0.13	2.15	1.26
Dorso-caudal pleurisy (score > 2), %	128	18	23.28	23.35	14.08	10.35	0.00	5.36	60.00	43.05

### Effect of tail caudectomy, season and distance to the abattoir on the body and tail lesions

The results of the statistical analysis on the effect of caudectomy, season and distance from the farm to the abattoir on body and tail lesions and respective LSI indices are shown in Table 3. Pigs with docked tails had a higher prevalence of score 2 lesions on ears (*P* = 0.015) and higher LSI of ears (*P* = 0.031) compared with undocked tails. The prevalence of lesions with scores 1 and 2 and the LSI of forequarter were significantly higher in batches of pigs with docked tail compared with undocked (*P* = 0.043; *P* = 0.003; *P* = 0.002; respectively). The prevalence of lesions with score 2 and LSI of the tails were significantly higher in batches of pigs with undocked tails compared with docked tails (*P* < 0.001).

**Table 3:** Effect of caudectomy, season and distance from farm to abattoir on lesions and lesion score index on tail and body of the carcass of heavy pigs

Item	Tail		SE	Season		SE	*P* value		
	Docked	Undocked		Warm	Cold		Tail	Season	Km abattoir
N batches*	143	28		89	82				
Lesions ears 0, %	89.80	93.70	1.47	87.60	95.90	1.39	0.080	< 0.001	0.017
Lesions ears 1, %	6.34	4.75	1.23	8.89	2.20	1.16	0.395	< 0.001	0.059
Lesions ears 2, %^3^	3.85	1.53	0.63	3.47	1.91	0.59	0.015	0.028	0.053
LSI ears^1,2^	14.04	7.81	1.90	15.83	6.01	1.80	0.031	< 0.001	0.012
Lesions forequarter 0, %	80.60	87.90	1.59	79.90	88.70	1.50	0.003	< 0.001	0.857
Lesions forequarter 1, %	11.21	8.31	0.94	12.71	6.81	0.89	0.043	< 0.001	0.172
Lesions forequarter 2, %^4^	8.17	3.75	0.99	7.43	4.49	0.94	0.003	0.009	0.322
LSI forequarter^1,2^	27.60	15.80	2.48	27.60	15.80	2.34	0.002	< 0.001	0.782
Lesions trunk 0, %	81.90	85.20	1.34	78.50	88.70	1.27	0.106	< 0.001	0.043
Lesions trunk 1, %	12.00	10.50	0.96	14.56	7.89	0.91	0.292	< 0.001	0.003
Lesions trunk 2, %	5.83	4.40	0.74	6.68	3.56	0.70	0.205	< 0.001	0.755
LSI trunk^1,2^	23.70	19.30	1.98	27.90	15.00	1.87	0.145	< 0.001	0.226
Lesions hindquarter 0, %	92.50	94.10	0.78	90.60	96.00	0.73	0.193	< 0.001	0.044
Lesions hindquarter 1, %	5.40	4.74	0.62	7.01	3.13	0.58	0.477	< 0.001	0.029
Lesions hindquarter 2, %	2.07	1.27	0.35	2.45	0.89	0.33	0.132	< 0.001	0.562
LSI hindquarter^1,2^	9.55	7.28	1.04	11.90	4.92	0.98	0.150	< 0.001	0.092
Lesions limbs 0, %	98.94	98.46	0.24	98.28	99.12	0.22	0.189	0.002	0.002
Lesions limbs 1, %	0.77	1.16	0.22	1.40	0.53	0.21	0.262	< 0.001	0.119
Lesions limbs 2, %	0.28	0.38	0.09	0.30	0.36	0.08	0.420	0.484	< 0.001
LSI limbs	1.33	1.92	0.28	1.99	1.26	0.26	0.162	0.020	< 0.001
Lesions tail 0, %	70.33	6.64	2.15	34.10	42.90	1.62	< 0.001	< 0.001	0.379
Lesions tail 1, %	23.00	26.60	1.91	27.10	22.50	1.80	0.223	0.032	0.220
Lesions tail 2, %	7.17	66.61	1.92	39.60	34.20	1.82	< 0.001	0.013	0.043
LSI tail^1,2^	37.40	159.80	3.75	106.30	90.90	3.54	< 0.001	< 0.001	0.147

*The number of animals per batch was around 100

^1^LSI: lesion score index

^2^Values are calculated on a range from 0 to 200 considering the prevalence and severity of lesions, where 0 is absence and 200 of all carcasses in the batch show severe lesions

^3^The interaction between tail and season was significant (*P* = 0.034). The interaction between docked tail and warm season shows higher percentage of lesions than intact tail and warm season (*P* = 0.008). The interaction between docked tail and warm season shows higher percentage of lesions than docked tail and cold season (*P* = 0.025)

^4^The interaction between tail and season was significative (*P* = 0.023). The interaction between docked tail and warm season shows higher percentage of lesions than intact tail and warm season (*P* = 0.001). The interaction between docked tail and warm season shows higher percentage of lesions than docked tail and cold season (*P* = 0.006)

In all parts of the body analyzed, the lesions were significantly higher in the warm season (between April and September) than in the cold season (between October and March) (*P* < 0.001).

The increased distance between farm and abattoir increased the ears lesion scored 0 (coef. = 0.024, *P* = 0.017), and ears LSI decreased with the increase of km to the abattoir (coef. = − 0.032, *P* = 0.012). On the contrary, the prevalence of lesions scored 0 in the trunk and hindquarter decreased in longer transports (trunk: coef. = − 0.018, *P* = 0.043; hindquarter: coef. = − 0.011, *P* = 0.044), while the prevalence of score 1 in the trunk and hindquarter increased with the increase of km to the abattoir (trunk: coef. = 0.019, *P* = 0.003; hindquarter: coef. = 0.009, *P* = 0.029). The prevalence of lesions scored 0 on limbs decreased with the km from the abattoir (coef. = − 0.005, *P* = 0.002), while the prevalence of lesions scored 2 on limbs and the limbs LSI were higher with the increase of km to the abattoir (coef. = 0.003, *P* < 0.001; coef. = 0.008, *P* < 0.001; respectively).

### Effect of tail caudectomy, season and distance to the abattoir on the lung injury

The effect of caudectomy, season and distance to the abattoir on lung injury is shown in Table 4. The caudectomy, season and length of the transport did not influence the prevalence of pulmonary lesions and pleuritis.

**Table 4:** Effect of tail caudectomy and season on indices and prevalence of lung lesions and pleuritis

Item	Tail		SE	Season		SE	*P* value		
	Docked	Undocked		Warm	Cold		Tail	Season	Km abattoir
N batches*	128	19		70	77				
Madec	0.89	0.90	0.13	0.89	0.89	0.12	0.989	0.956	0.372
EP lung lesions (%)	28.00	26.64	2.45	25.70	28.80	2.28	0.742	0.221	0.689
SPES	0.79	0.83	0.07	0.82	0.80	0.06	0.703	0.794	0.142
Pleurisy, %	36.20	40.10	2.72	38.30	37.90	2.53	0.356	0.878	0.139
APP index	0.66	0.66	0.07	0.68	0.65	0.06	0.964	0.646	0.120
Dorso-caudal pleurisy (score > 2), %	23.40	22.90	2.22	23.30	22.90	2.06	0.885	0.839	0.091

*The number of animals per batch was around 100

### Effect of tail caudectomy, season and distance to the abattoir on the carcass quality

The effect of caudectomy, season and distance of the farms to the abattoir on hot carcass weight, percentages of lean meat in the carcass and carcasses included in the PDO classification is shown in Table 5. The caudectomy of the tail influenced the carcass weight and the percentage of lean meat in the carcass. The average hot carcass weight was higher in batches with docked tail than in batches with undocked tails (*P* = 0.027), while the percentage of lean meat was higher in batches with undocked tails compared to batches with docked tails (*P* < 0.001). The percentage of carcasses included in the PDO was significantly higher in batches with docked tails compared with undocked tails (*P* < 0.001) and in the cold season compared with the warm season (*P* < 0.001). The length of the transport influenced the percentage of lean meat of the carcass and the percentage of carcasses included in the PDO; in detail, the percentage in lean meat increased with longer transports (coef. = 0.005, *P* < 0.001) and the percentage of carcasses included in PDO (coef. = − 0.0003; *P* = 0.002).

**Table 5:** Effect of tail caudectomy and season on weight, percentage of lean meat and the percentage of carcasses included in the PDO classification

Item	Tail		SE	Season		SE	*P* value		
	Docked	Undocked		Warm	Cold		Tail	Season	Km abattoir
N batches*	143	28		89	82				
Average hot carcass weight, kg	142.00	139.00	0.90	140.00	141.00	0.85	0.027	0.123	0.297
Average % lean meat	52.18	53.26	0.18	52.90	52.50	0.17	< 0.001	0.063	< 0.001
No PDO carcasses, %	21.30	31.20	1.69	29.60	22.80	1.59	< 0.001	< 0.001	0.002
PDO carcasses, %	78.70	68.80	1.69	70.40	77.20	1.59	< 0.001	< 0.001	0.002

*The number of animals per batch was around 100

### Correlations between lesions and carcass quality parameters

A negative correlation between the percentage of carcasses included in PDO and the LSI of limbs (r = − 0.315; *P* < 0.001) and with the LSI of tails (r = − 0.422; *P* < 0.001) was observed. Additionally, a positive correlation was found between the percentage in lean meat and the LSI of tails (r = 0.338; *P* < 0.001).

Considering exclusively the batches with undocked tail, a negative correlation between the percentage of carcasses included in PDO and the LSI of trunks (r = − 0.50; *P* = 0.01), the LSI of tails (r = − 0.045; *P* = 0.03) and the lesions of score 2 of tails (r = − 0.50; *P* = 0.01) was observed. Additionally, a positive correlation was found between average of lean meat of the carcass and the LSI of tails (r = 0.48; *P* = 0.02). Considering exclusively the batches with docked tails, there was a negative correlation between the percentage of carcasses included in PDO and the LSI of tails (r = − 0.2; *P* = 0.002) and the lesions of score 2 of tails (r = − 0.34; *P* < 0.001).

No significant correlations between all body skin lesions and pulmonary lesions and pleuritis were found, regardless of docking status.

## Discussion and conclusions

In the present study the lesions have been evaluated in the abattoir, which means that they could occurred during the breeding phase, the transport and/or at the unloading of animals at the abattoir.

The present study showed that the prevalence of lesions on tail, trunk and forequarter was higher than in the other parts of the body, and limb was the part with the lowest prevalence of injuries. The present results partially agree with the study by Driessen et al. [18], in which a higher prevalence of skin lesions can be observed in the shoulder region of pig carcasses at the abattoir (31%), compared to the other parts of the body. Lesions can be due to fighting and bites which can be targeted to ears, neck, face and shoulder [18]. Besides the fighting and bites at the farm, lesions on carcasses can also be due to fighting during the transport to establish dominance, to the driving style [18] and to the time that animals rest in the lairage [19]. Anyway, with the observation on the slaughter line is not possible to discern the timing of occurrence of the lesions. Regarding the tail lesions in the present study, the average means of tails without lesions was 70.44% in pigs with docked tails and 4.96% in pigs with undocked tails. An appropriate comparison between the present results and the percentages of tail lesions in Europe is difficult to be done. In Switzerland, where tail docking was prohibited in 2008, a study of 2021 showed that 63% of the examined carcasses had an intact tail tip while 37% had non-intact tail tip [20]. Similarly, in Finland, 49.2% of all tails were classified as completely intact [21]. Martinez et al. [22] reported that in Spain 2.9% of the condemned carcasses had tail lesions.

It is widely argued that, in the current intensive pig production systems, stress conditions can shift normal behaviour into abnormal behaviour [5, 6, 8]. Pigs without exploratory material become restless and redirect their exploratory behaviour such as rooting and chewing to the tails and ears of pen mates [5, 23]. For these reasons, the observation of tail lesions at the abattoir can be considered an indicator of stress and a poor rearing environment. In fact, tail lesions are being considered by other authors as an iceberg indicator of animal welfare in the farm [24]. Furthermore, the European Union, trying to answer to public demands for the increasing concerns regarding animal welfare, by providing the Recommendation (EU) 2016/336 [25], regarding the minimum standards on laying down for the protection of pigs to reduce the need for tails docking. However, in Italy, data regarding pigs rearing with docked tails are still poor. Indeed, in Italy, the first actions taken to reduce routine tails docking just started in 2019 with the publication of the National Plan, which obligates farmers to rear pigs with undocked tails starting with the introduction of 3% of undocked tails pigs [26].

In this study, tail lesions were higher and more severe in batches with undocked tails than in batches with docked tails. The results obtained agree with several studies [5, 27, 28]. Although the lower stocking density, provision of sufficient feeding space, no fully slatted floor and the regular provision of enriched materials are effective to reduce tail biting and lesion in undocked pigs [29], tail docking is still an effective management procedure able to reduce the tail biting [27], therefore caudectomy of the tail is commonly used as a preventive measure to reduce this problem [5, 28]. Although there are several arguments against tail docking, for instance, the pain, which can be caused by neuroma formation, indicating an increased sensitivity to pain at the amputation point, or the risk of infection [8, 30]. Another important argument is the increase in piglets’ stress due to the amputation, demonstrated by the increase in cortisol [31, 32]. On the other hand, several studies have evidenced that tail biting is a behavioural problem with a multifactorial origin and reported other different management measures important for reducing tail biting lesions [33–35]. Some factors mostly related to tail biting included the absence of exploratory material like straw, which allows rooting, a natural behaviour of pigs, the presence of slatted floors and a barren environment [5]. Other important category factors influencing the occurrence of tail biting are related to the environment where animals live, which include temperature, humidity, light, space allowance and others, but also feed and water availability and feed type are important factors [34, 35].

In the present study, results showed that pigs with docked tails have a higher frequency of lesions on the ear and forequarter than undocked pigs. The higher tendency of ear biting in pigs with docked tails is sustained by other authors [36, 37]. This could be explained by the fact that in tail docking swine population, the biters pigs can redirect their attention from tail to ears, legs, or other parts of the body of their box mates, probably because they became more likely to bit and more aggressive [36, 38, 39], or because of the formation of neuromas which increase the sensitivity to pain in docked tail animals [30].

Among the different factors that can affect carcass lesions, the duration of transport has been indicated as one of the main ones [7, 40]. Skin lesions inflicted in the last 48 h of life are the most frequently observed at abattoirs and have an impact on meat quality [7, 40].

In the present study, it was found that body lesions, especially of ears, limbs and tail were higher when transport was longer. This result agrees with previous studies. For instance, Mota-Rojas et al. [41] found that transport time affected the incidence of general skin lesions and bruises and that long transport can reduce swine carcass yield [41, 42], probably because animals drink more water in long transport, therefore this implies a higher liquid loss during the evisceration [41]. The presence of trauma can be related to falling and balance loss during transport, which can be associated with the truck structure, density and with the way the driver controls the vehicle especially when it stops, and it can be related to the loss of weight by condemning the injured tissue [41, 43]. The longer is the journey, the greater is the probability that animals will injure themselves [41, 43].

The season was significantly associated with lesions in the present study, and particularly, it has been observed that lesions in all parts of the carcasses were significantly higher in the warm season. In this regard, literature reports discordant data [6, 7, 13, 44]. The no consensus on seasonal effect on body lesions could be due to the fact that aggressive behaviours between pigs might occur when animals are stressed by the temperature whether it is too high or too low [8]. In addition to the season and the temperature, ventilation methods in the farm as well as during the transport can also influence the state of the animal and therefore the occurrence of lesions. Contrary to this study, Scheeren et al. [44], sustained that cold temperatures contribute to the increase in bruised carcasses. Similarly, Bottacini et al. [6] found that winter and spring were the seasons with the highest ear lesion frequency. While other authors report higher body lesions and fighting-type bruises in pigs transported to slaughter during summer compared to winter [7, 10]. The greater proportion of lesions during summer is hard to explain considering that pigs lie down more during the transport compared to winter, but Torrey et al. [9] reported that during the transport pigs experienced more falling, slipping, overlapping, and walking backwards during summer than in winter.

The pigs monitored during this study showed a prevalence of bronchopulmonary lesions of 27.32%. This data is lower compared to the results of a study conducted in 2008 [45] in Italy, confirmed by Pangallo et al. [46], and recently updated by Vitali et al. [47], in 2021, in which the prevalence of EP like lesions, was 46.4%, 46% and 30.2% respectively. Regarding the severity of the lesions observed, a further decrease in the Madec mean of 0.89 is reported in the present study, when compared to what was reported in the same studies, where the EP like lesions average value was 1.03, 1.09 and 0.91, respectively. The prevalence and EP like lesions average value data confirm what was observed by Vitali et al. [47], providing an updated view on the monitoring of enzootic pneumonia in pigs reared in Italy. An interesting aspect is the percentage of pleurisy, which in the present study was at 38.29%. This data also appears reduced when compared with the results obtained in the years 2008–2011, with values of 47.5%, 42.5% respectively [45, 48] and in line with those reported by Vitali et al. [47] in 2021 (38.4%). Regarding the detection of pleuritis, the data obtained indicate the presence of dorso-caudal pleuritis (score ≥ 2) is in line with previous data of 25.1% reported in Italy by Merialdi et al. [45] for the year 2008, with the 24.2% reported in Luppi et al. [48] for the year 2008–2011 and with the more recent data reported by Vitali et al. [47] of 25.7%. Since no information regarding the health status, including the use of vaccination against *Actinobacillus pleuropneumoniae*, are available, it is not possible to make a conclusive assessment of the reasons for the reduction in the EP like lesions. Therefore, results in EP like lesions remain descriptive of the current general situation.

In the present study the caudectomy, the season and the length of the transport did not influence the prevalence of pulmonary lesions and pleuritis. The nonsignificant effect of the season on pulmonary lesions and pleuritis is confirmed by Vitali et al. [47].

No effect of tail caudectomy on pulmonary lesions and pleuritis was also confirmed by Kritas et al. [49], however, other authors reported an association between tail length, body lesions and lung lesions or pleurisy suggesting the existence of a relationship between poor health and poor welfare of pigs [15, 17]. Factors that may differ between countries and studies are several, for instance, the management of the farm, the genetics of the animals, general health status of the herd. Moreover, the currently available data are insufficient to provide definite conclusions.

This study reports that the weight of carcasses was lower, and the percentage of lean meat was higher in pigs with undocked tails compared to pigs with docked tails. It has been suggested by other authors that higher percentage of lean meat and a lower backfat thickness were associated with the higher prevalence of tail biting [50, 51]. Moreover, in agreement with the data reported by Valros et al. [52], stressed animals, including pigs with bitten tails, had a lower carcass weight and produced a lower total amount of lean meat, but had a higher percentage of lean meat than non-stressed pigs. This result is relevant for the Italian pig production chain, characterized by the heavy pig, where carcass fatness is of key importance for the meat quality. In addition, the present study also suggested that docked tail batches had a higher percentage of carcass included as PDO than batches with undocked tails. Furthermore, limbs and tail lesions were positively correlated to a lower percentage of PDO carcasses. Performing the correlations in batched with docked and undocked tails separately, a negative correlation between tail lesions and the percentage of PDO carcasses was observed in the long tail. There is no other similar data in the literature to explain these findings.

In conclusion, this study provided the widest published dataset referring to the comparison between docked and undocked tail pigs, providing updated data and benchmarking for future studies. From the practical point of view, considering the low proportion of undocked pigs compared with the docked pigs in the Italian farms, this study highlighted the need to improve the rearing management to minimize the lesions in the undocked tails pigs While our dataset does not confirm the correlation between tail lesions and respiratory disease. On the other hand, the positive correlation between tail lesions and the exclusion of the fresh ham to the PDO system poses the urgency to improve the rearing system and to manage this issue, especially for the Italian pig production system which is based on the PDO cured ham in order to have a high economic impact for the whole sector.

## Methods

The study was based on the integrated evaluation method developed in the RDP Emilia-Romagna Focus area 3A-Operation 16.01.1 (WELDONEPIG) project [7], which is briefly described in the present section. This study was conducted between June 2019 and March 2022 for a total of 21 days of observation, 10 days during the cold season and 11 during the warm season. The inspections were carried out in two Italian abattoirs, and a total of 171 batches were examined from 103 different farms, 82 batches during the cold season and 89 during the warm season. About 8 batches for day and around 100 animals for each batch were evaluated. Of these batches, 143 (14.403 animals) were tail docked and 28 (2.853 animals) had  undocked tail for a total of 17.256 carcass from 102 herds randomly selected from abattoir providers.

The inspections were carried out by trained personnel involved in the project during different seasons and were classified as the warm season (temperature between 16 °C and 31 °C): between April and September and the cold season (temperature between 5 and 19 °C): between October and March.

The tail lesions and body and bronchopulmonary and pleural lesions were evaluated during each inspection day. In particular, tail and body lesions were assessed in all animals and batches counted, while bronchopulmonary and pleural lesions were assessed for 148 batches, 128 with tail docked (12.131 animals) and 19 with intact tail (1.812 animals), for a total of 13.943 animals. Tail and body lesions were assessed following the recommendations of the Welfare Quality protocol (2009).

Briefly, the tail lesion scores ranged from 0 to 2, 0 = no injuries; 1 = superficial bite along the tail caudectomy but no evidence of swelling; 2 = visible open lesion on the tail, presence of scarring, swelling or partial absence of the tail. The scores for injuries to the tail were assessed by one trained person staying on a raised floor at the end of the dressing line, where it was possible to observe the tails closely. For body injuries, one side of the carcass was evaluated by another trained person on the dressing line (after the veterinary inspection point) and injuries were scored in 5 separate areas (ear, forequarter, trunk, hindquarter and limbs). The score was: 0 = up to 4 visible lesions; 1 = 5 to 10 visible lesions; 2 = 11 to 15 visible lesions. All the evaluations were performed immediately after the carcass evisceration.

The results of each batch were expressed as the prevalence of the scores obtained (0, 1, 2) considering the total observations of a batch. A lesion score index was then calculated, which considered both the frequency and the gravity of the lesions (in a range from 0 to 200, where 0 is absence and 200 is all animals with severe lesions), it was calculated as follows [7]:

$$Lesion\;score\;index{:}\;[\% \;lesion\;type\;1 + \left( {2*\% \;lesion\;type\;2} \right)]$$

Lung scoring was performed by a veterinarian of the Experimental Zoopropfylactic Institute of Lombardy and Emilia Romagna (IZSLER). The bronchopulmonary lesions suggestive of Enzootic pneumonia like lesions (EP like lesions) were scored from 0 to 4 for each lobe (0 = no lesion; 4 = lung lobe lesions affecting an area > 75%), up to a maximum score of 28, according to the Madec et al. method [53] (0 = absence of lesions; 1 = lesions in < 25% of the lobe; 2 = lesions in 26–50% of the lobe; 3 = lesions in 51–75% of the lobe; and 4 = lesions in > 75% of the lobe) (Additional file 1: Figure S1). Finally, the EP like lesions average value per batch was calculated (sum of the average score of each lobe/number of lungs examined).

Pleurisy was scored using the SPES grid (*Slaughterhouse pleuritis evaluation system*) considering a 0–4 scale depending on the extension and the location of the pleuritis, according to the method devised by Dottori et al. [54], briefly: 0 = absence of lessons from chronic pleurisy; 1 = antero-ventral lesions: pleural adhesions between lobes or at ventral lobe borders; 2 = unilateral dorso-caudal focal lesions; 3 = bilateral type 2 lesions or extensive unilateral lesions (at least 1/3 of a diaphragmatic lobe); 4 = severe bilateral extensive lesions (at least 1/3 of both diaphragmatic lobes). The SPES grid provides two results: the average SPES value (sum of the individual pleuritic scores/number of lungs evaluated), which describes the general degree of pleurisy of the batch, and APPI index, which provides information on the prevalence and severity of dorso-caudal pleuritis, which is strongly correlated with previous *A. pleuropneumoniae* infections. The APPI index is calculated by applying the following equation: frequency of dorso-caudal lesions (scored 2, 3 and 4) multiplied by the mean calculated considering only lungs with dorso-caudal lesions (scored 2, 3 and 4).

The carcass quality data were provided from the abattoir and were the hot carcass weight, measured during the slaughter chain after the evisceration, and the % of lean meat, obtained by measurement using Fat-o-Meater (OM-SFK, Copenhagen, Denmark) placed on the slaughter chain. The results are expressed as the average of a single batch. In addition, the abattoir provided the SEUROP classification, representing muscle percentage and fat deposit classes, and the weight category base on the carcass weight (Light: 70–110 kg or Heavy: > 110 kg) of each carcass [55]. With these last parameters, we calculated the percentage of carcasses accepted as PDO (Protected Designation of Origin) for each batch, thus, heavy carcasses with a U, R or O classification, according to the product specification of the PDO "Prosciutto di Parma" [56]. In addition, the abattoir provided us the distances in km of the farms from the abattoir.

### Statistical analyses

Statistical analyses were carried out using Excel and R software (R Core Team, 2017). The batch was used as the statistical unit. Data were analyzed using a General Linear Model (GLM) in which tail caudectomy (docked or undocked), slaughter season (warm or cold) were included as fixed factors and distance (km) between farm and slaughtering was included as covariate. Interaction between tail caudectomy and season was tested and removed when resulted not significant. Values with a *P* ≤ 0.05 were considered significant while a 0.10 ≤ *P* > 0.05 was considered a trend. In addition, Pearson correlations between carcass quality parameters (weight, % of lean meat and PDO classification) and lesions parameters and pulmonary lesions and pleuritis percentage in the carcass were analyzed using the “Hmisc” package in R software.

## Supplementary Information

**Additional file 1. Supplementary Figure 1.** Examples of the lesions distribution related to the lobe scores Diagram of pig lungs: dorsal view. A. right apical lobe; B. right cardiac lobe; C. right diaphragmatic lobe; D. azygos lobe; E. left diaphragmatic lobe; F. left cardiac lobe; G. left apical lobe.

## Data Availability

Not applicable.

## Abbreviations

LSI: Lesion score index
APPI: *Actinobacillus pleuropneumoniae* index
SPES: Slaughterhouse pleuritis evaluation system
PDO: Protected Designation of Origin
GLM: General linear model
