# Supplementary material for: Evaluation of carcass quality, body and pulmonary lesions detected at the abattoir in heavy pigs subjected or not to tail docking
Source: Porcine Health Manag. 2023 Feb 15;9:4. doi: 10.1186/s40813-022-00297-4 (PMC9930331; doi:10.1186/s40813-022-00297-4)
Supplement: Supplementary file 1 — Additional file 1. Supplementary Figure 1. Examples of the lesions distribution related to the lobe scores Diagram of pig lungs: dorsal view. A. right apical lobe; B. right cardiac lobe; C. right diaphragmatic lobe; D. azygos lobe; E. left diaphragmatic lobe; F. left cardiac lobe; G. left apical lobe. [file 40813_2022_297_MOESM1_ESM.pdf]

Evaluation of carcass quality, body and pulmonary lesions detected at the abattoir in heavy pigs subjected or not to tail docking

[1]Laura Amatucci, [1]Diana Luise, [2]Andrea Luppi, [1]Sara Virdis, [2]Alice Prosperi, [1]Agatha Cirelli, [2]Claudia Bosco and [1]Paolo Trevisi

Affiliation: [1]*Department of Agricultural and Food Sciences (DISTAL), Alma Mater Studiorum - University of Bologna, Viale G. Fanin 46, 40127 Bologna, Italy*

[2]*Istituto Zooprofilattico Sperimentale della Lombardia e dell'Emilia Romagna (IZSLER) "Bruno Ubertini"-Via Bianchi, 9-25124 Brescia*

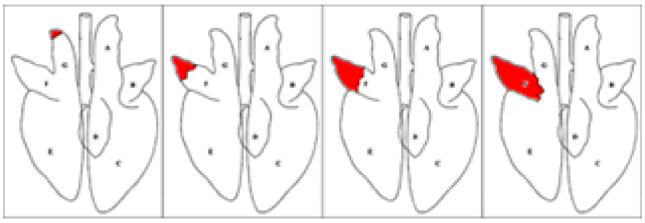

Supplementary Figure 1. Examples of the lesions distribution related to the lobe scores

Diagram of pig lungs: dorsal view. A. right apical lobe; B. right cardiac lobe; C. right diaphragmatic lobe; D. azygos lobe; E. left diaphragmatic lobe; F. left cardiac lobe; G. left apical lobe.
